# Status and predictors of medical students’ knowledge and attitude towards palliative care in Jordan: a cross-sectional study

**DOI:** 10.1186/s12904-023-01338-7

**Published:** 2024-01-03

**Authors:** Wejdan Y. Younis, Ayman M. Hamdan-Mansour

**Affiliations:** 1 Clinical Nursing Department, School of Nursing, The University of Jordan, Amman, 11942 Jordan; 2 School of Nursing, The University of Jordan, Amman, 11942 Jordan

**Keywords:** End-of-life care, Knowledge and skills, Medical students, Palliative care

## Abstract

**Background:**

Palliative care focuses on the ability of medical students to use their expanded experiences and knowledge; however, basic medical education does not provide adequate knowledge and skills regarding palliative and end-of-life care. This study designed to examine knowledge related to palliative care and attitudes toward dying people among medical students in Jordan.

**Methods:**

Cross-sectional, descriptive design was used in this study. A total of 404 medical students were recruited using convenience sampling techniques from six medical programs. Data was collected using a self-administered questionnaire in relation to knowledge and attitudes regarding palliative care and dying persons using Palliative Care Assessment Knowledge (PCAK) and Frommelt Attitudes toward Care of the Dying Scale Form B (FATCOD-B).

**Results:**

Medical students have a moderate level of knowledge related to palliative care in (PCAK) part1 subscale about; pain managing (*n* = 156, 38.6 %), managing other palliative care symptoms (*n* = 164, 40.6 %), and in witnesses' family counseling and breaking bad news discussion (*n* = 178, 44.1 %). However, medical students have inadequate knowledge concerning the palliative care in part-2of the scale. Furthermore, students have positive attitudes towards caring for dying patients with mean score of 108.76 (SD = 8.05). The highest ranked attitude subscales were; Fairs/Malaise (*M =* 29.03, SD = 4.28), Communication (*M = 21.39*, SD = 2.51) and Relationship (*M = 18.31*, SD = 1.55). There was a significant difference in attitudes in relation to gender (*t* = -5.14, *p* < 0.001) with higher female mean score (M = 109.97) than males (M = 105.47). Also significant difference found among those who are exposed to Palliative Care and those who are not (*t* = -6.33, *p* < 0.001) with higher mean score of those exposed to palliative acre courses (M=298) than those who did not (M=106).

**Conclusion:**

Improving knowledge and attitudes of medical students regarding palliative and end of life care should be highlighted to medical educators and medical schools need to incorporate palliative and end-of-life care into medical curricula across all levels

## Introduction

The World Health Organization (WHO) estimated that non-communicable diseases (NCDs) cause death to 41 million people each year which is equal to 71% of all deaths, worldwide [1]. This might warrant healthcare professionals to integrate palliative care in health care system and in academic institutions into their curricula. It is assumed that healthcare systems are responsible for demonstration of appropriate palliative care to improve quality of life for patients and their families’ facing life threatening conditions such as cancer [2, 3]. Therefore, palliative and end of life care are genuinely proposed to healthcare service providers to ensure positive healthcare outcomes. Although topics related to oncology and palliative care considered significant ones in medical curricula, the literature shows that such topic is not receiving the attention and concerns of medical educators [3]. This would provoke attention and raise questions regarding integrating palliative care in medical education.

Among medical students, the effectiveness of palliative and end of life care underscore the importance of undergraduate palliative care curriculum in medical schools [4, 5]. It has been reported that medical students exposed to palliative care training in their curriculum showed improved self-confidence and higher levels of concern and competency in palliative care services [4, 5]. Higher levels of knowledge, improved communication, and personal development have been reported among medical students exposed to palliative care courses [5]. This may urge the need to introduce palliative and end of life care early into medical education curricula. With acceleration and development of technology in medical education, integrating palliative and end of life care would be one step toward ensuring competency of medical students in the field. Such a step would enable medical students to recognize the importance of palliative and end of life care and warrant medical educators to the need of such integration in medical education as part of their role [6, 7].

In Jordan, similar to other Arab and Muslim countries, have a particular belief and traditions about death and dying [8]. The traditions and cultural role in defining death and care of dying individuals do influence attitudes and practices of healthcare professional, in particular, medical team. The medical education; therefore, need to be tailored to correspond to beliefs and values of the Jordanian society. This would also indicate the need to have medical students consider to integrate the Arabian culture related to death and dying into their practices. However, if medical students are not prepared theoretically and clinically to provide palliative and end of life care, this would affect their practices as students and as physician and specialized personnel in the future. They would rely on their personal acquired knowledge which may affect and create inconsistent treatment plans, and consequently, affecting the quality of care outcomes. Their attitudes and knowledge will also guide their decisions regarding plans of care of their patients [9].

To the authors' knowledge, and especially among the Arab culture, this topic has never been addressed in terms of medical students' knowledge towards palliative care and attitudes regarding caring for dying patients. Shortly, the lack of curricular emphasis and influence of culture on medical practice related to end of-life care necessitate this study. This study examines the knowledge and attitudes toward dying people among medical students.

The specific aims of this study are:

- To examine the status of knowledge and attitudes of medical students toward end-of-life care
- To test the relationship between attitude and socio-demographic characteristics of students.

## Methodology

### Study design

Descriptive cross-sectional design was utilized. Data was collected from medical students using self-administered questionnaires regarding knowledge and attitudes towards caring for dying patients. Data collection and analysis has been in line with ethical and scientific guideline at the University of Jordan.

### Settings

The study conducted at universities that offers medical education. In Jordan, there are 12 publics and 24 private universities; however, only six universities have schools of medicine. The participants were selected using a convenience sampling technique from the six selected universities and those are: The University of Jordan, Applied Balqaa University Jordan Jordan University of Science and Technology, Hashemite University, Mutaa University, and Alyarmook University. Medical education in Jordan has been established in 1971 at the largest leading university in Jordan; the university of Jordan. The schools offer the "Doctor of Medicine" curriculum that is a six-year undergraduate degree. All medical programs adhere to Ministry of Higher Education (MOHE) guidelines with very minimal differences. The core courses are the same, while differences made in the elective university courses. This has caused minor difference in the number of credit hours of the programs. All universities follow the same path of education and clinical training throughout the six years where students start their clinical training at the first semester of the 4^th^ year. None of the schools offer end of life care or palliative; however, students are enrolled in oncology training and theory preparation that embeds palliative and end of life care.

### Sample and sampling

A convenience sampling technique was used to recruit medical students. The inclusion criteria were: 1) be a regular student at the university, and 2) has finished at least one semester in clinical training which means the students has to be at the fourth year level to ensure that the students have completed the three years theory preparation. No exclusion criteria were used to maximize participation. The sample size was determined using a G* Power 3.1 [10] for the independent t – test, at .05 two tailed level of significance, power of .80 (1- β = .20), moderate effect size of 0.3. The estimated sample size was at least 352 students.

### Data collection method

Prior data collection, ethical approval was obtained from the targeted universities. The universities then communicated to facilitate approaching students at medical schools. The university facilitator role limited to enable announcing the study and it purpose to the students through students' board and social media groups. Those interested were asked to contact the researcher who have explained the study purpose, significance, and ensured the participants that their participation is voluntary and they have the right to refuse participate without any direct or indirect influence on their status as medical students. The researcher also addressed anonymity, privacy and confidentiality of the study and the students have the right to withdraw from the study at any time. The students were then provided with consent forms to be signed with a code per student to avoid disclosure of the information. The students were directed where to return the survey and have been provided with sealed envelopes and asked to drop the survey at a box at the research facilitator's office at their convenience. The survey took 30-45 min in average. Data has been collected at the period prior to final examinations where student can take few days to return the survey. The research team who have assisted in data collection were independent researchers and not employed by any of the universities. The soft copy of the data saved to password protected computer and the hard copy of study materials and questionnaires were kept at locked cabinet the researchers' office.

### Instrument

To study medical students' attitudes, and knowledge toward the care of dying patients, the following tools was utilizing;

- Frommelt Attitude Toward the Care of the Dying Scale Form B (FATCOD-B) (Frommelt, 2003) [11]. The (FATCOD) is the only psychometric assessment tool that specially [12] in detecting attitudes towards caring for dying patients among students at health school [13]. Additionally, the tool was confirmed by research its validity and reliability, and considered as one of the most commonly utilize tool to assess attitude of those undergraduate medical students [14–16].
- Palliative care assessment knowledge (PCAK) [17]. The tool was developed in (2019) to assess non palliative physician knowledge regarded palliative care, and (PCAK) meets psychometric criteria for reliability and construct validity. It contains of 2 parts; the first part was about the self-reported knowledge using also 5 points Likert scale. The second part is 12 clinical questions it is multiple choice questions, the correct answer was considered as “True” and all other answer “false” [17, 18].

In addition, an author-demographics section has also been provided to collect data regarding age, gender, academic year, palliative care training, etc.

### Ethical consideration

Different steps and measures was used instituted to guarantee the human rights of the study participants. The study will be designed to ensure the ethical principles of; non-maleficence “no harem”, voluntary participation, confidentiality, and the right to drop from the study at any point without any consequences. Moreover, IRB approval was obtained from the ethics and research committee at School of Nursing at the University of Jordan. In addition, approval for participation obtained from all other participating universities.

### Data analysis

The data analyses were performed by using IBM-SPSS version 25. The data was analyzed as reported. Descriptive statistics including central tendency measures (Mean and Median) and dispersion measures (SD, range, IQR) were used to assess the knowledge level of palliative care and attitude toward end of life care by medical students. Percentages and frequencies were also used to describe categorical variables. In addition, t-test for two independent samples were also used to examine the differences in attitudes and knowledge in relation to two-leveled variables, ANOVA for three or more level of measurement variables between certain socio-demographic. Alpha was set to .05 level of significance.

## Result

### Demographic characteristics of the study participants

The sample of the study consisted of Jordanian undergraduate medical students (*n*= 404). The analysis showed that (see Table 1) that the mean age of the students was = 23.4 (SD= 1.2) ranging from 20 to 26 years. Female students represented 73.0% (*n* =295), all of them were single (*n* =404, 100 %), and almost half of them were their 6^th^ year (*n*= 224, 55.4 %).

**Table 1:** Characteristics/demographics of the study sample (*n*=404)

**Variable**		**Frequency (%)**
**Gender**	**Female**	295 (73 %)
	**Male**	109 (27 %)
**Marital status**	**Single**	404 (100 %)
	**Married**	0 (0 %)
**Academic Years**	**5**^**th**^** year**	180 (44.6 %)
	**6**^**th**^** year**	224 (55.4 %)
**The Region of the University**	**The Northern district**	50 (12.4 %)
	**The Central district**	308 (76.2 %)
	**The Southern district**	46 (11.4 %)
**Exposure to Palliative Care**	**No**	106 (26.2 %)
	**Yes**	298 (73.8 %)
**Palliative Care Course**	**No**	394 (97.5 %)
	**Yes**	10 (2.5 %)
**Education about Palliative Care**	**No**	217 (53.7 %)
	**Yes**	187 (46.3 %)

Regarding to the palliative care, most of students reported being exposed to palliative care concepts at their programs (*n* = 298, 73.8 %), while 97.5% (*n* = 394) confirmed that they have not taken a distinct course titled palliative care nor particular education about palliative care (*n* = 217, 53.7 %).

### Knowledge level of the medical students regards palliative care

The analysis (see Table 2) of part-1 of the attitude and knowledge scale (PCAK) indicated that medical students have moderate level of knowledge in relation to palliative care. It has been noticed that responses to good and weak were almost equal and very close indicating that students wert not consistent and are not sure about their responses confirming the moderate level of knowledge. Individual item analysis showed that 38.6 % (*n* = 156) of the medical students have good knowledge about their experience in managing pain in cancer and palliative patients, and 40.6 % (*n* = 164) have good knowledge level about their experience in managing palliative care symptoms such as constipation, nausea and vomiting, and anorexia. The highest reported item was (*n* = 178, 44.1 %) related experience in witnessing family counseling and breaking bad news discussion.

**Table 2:** Knowledge level of the medical students: part 1 (*n*=404)

**Item**	**Frequency (%)**
**1. How would you rate your experience in managing pain in cancer and palliative patients?**	
**None**	105 (26 %)
**Weak**	143 (35.4 %)
**Good**	156 (38.6 %)
**Very Good**	0 (0%)
**Excellent**	0 (0%)
**2. How would you rate your experience in managing other palliative care symptoms (constipation, nausea and vomiting, anorexia, etc.)?**	
**None**	58 (14.4 %)
**Weak**	154 (38.1 %)
**Good**	164 (40.6 %)
**Very Good**	28 (6.9%)
**Excellent**	0 (0 %)
**3. How would you rate your experience in witnesses' family counseling and breaking bad news discussion?**	
**None**	38 (9.4%)
**Weak**	160 (39.6 %)
**Good**	178 (44.1 %)
**Very Good**	28 (6.9 %)
**Excellent**	0 (0 %)

Regarding part-2, the analysis (see Table 3) indicated that medical students have inadequate knowledge concerning the palliative care. The questions that was introduced as short exam regard palliative care, showed that most of the students fail to have the correct answers. The two most reported items as correct ones are: Question 1 “palliative care is different from traditional care because palliative care?” (*n* = 367, 90.8 %), question 2 “which of the following members of the healthcare team are important to the delivery of palliative care?” (*n* = 404, 100 %). While all other question had a correct answer of equal or less than 25%.

**Table 3:** Knowledge level of the Medical students: part 2 (*n*=404)

**Item**	**Frequency (%)**
**1. Palliative care is different from traditional care because palliative care:**	
**g) I don't Know**	
**h) Is curative.**	
**i) Is equivalent to hospice care.**	
**j) Is focused on comfort, rather than cure.**	
**k) Is equivalent to end of life care**	
**l) Withdraws care.**	
**False**	37 (9.2 %)
**True**	367 (90.8 %)
**2. Which of the following members of the healthcare team are important to the delivery of palliative care?**	
**g) I don't Know**	
**h) Physicians**	
**i) Nurses**	
**j) Dietitians**	
**k) Physical and occupational therapists**	
**l) All of the above**	
**False**	0 (0 %)
**True**	404 (100 %)
**3. Which of the following drugs is considered weak opioids?**	
**g) I don't Know**	
**h) Morphine**	
**i) Codeine**	
**j) Hydromorphone**	
**k) Fentanyl**	
**l) Oxynorm**	
**False**	291 (72 %)
**True**	113 (28%)
**4. Which is the most appropriate drug can be used in management of delirium in palliative care:**	
**a) I don't Know**	
**b) Fentanyl**	
**c) Haloperidol**	
**d) Midazolam**	
**e) Zofran**	
**f) Paroxetine**	
**False**	318 (78.7 %)
**True**	86 (21.3 %)
**5. Dyspnea related to advanced lung cancer can be best treated by:**	
**g) I don't Know**	
**h) Morphine**	
**i) Midazolam**	
**j) Dexamethazone**	
**k) Oxygen mask and Ventolin**	
**l) 1 & 2**	
**False**	315 (78 %)
**True**	89 (22 %)
**6. The second step in WHO ladder for treatment of chronic pain is:**	
**g) I don't Know**	
**h) Strong opioid ± adjuvant therapy**	
**i) Non-opioid ± adjuvant therapy**	
**j) Weak opioid ± adjuvant therapy**	
**k) Adjuvant therapy**	
**l) Strong opioid ± pain intervention modalities**	
**False**	225 (55.7 %)
**True**	179 (44.3 %)
**7. Regarding Hypercalcemia in cancer patients, it:**	
**g) I don't Know**	
**h) Is the most common life threating metastatic disorder in cancer patients**	
**i) Needs to be corrected if adjusted serum calcium ≥3.5 mmol/l or the patient is symptomatic.**	
**j) Related to bone cancer only.**	
**k) Can cause severe diarrhea.**	
**l) All of the above**	
**False**	374 (92.6 %)
**True**	30 (7.4 %)
**8. The hallmarks of opioid toxicity are all the following except:**	
**g) I don’t Know**	
**h) Oxygen saturation ≤ 90%**	
**i) Respiratory rate ≤ 10 /min**	
**j) Pinpoint pupils**	
**k) Sedation**	
**l) Jerky movement**	
**False**	352 (87.1 %)
**True**	52 (12.9 %)
**9. Signs of superior vena-cava obstruction are all the following *EXCEPT*:**	
**g) I don't Know**	
**h) Cyanosis**	
**i) Pulsatile distended neck veins**	
**j) Edema in the hands**	
**k) Dilated veins over the chest wall**	
**l) Periorbital edema**	
**False**	363 (89.9 %)
**True**	41 (10.1 %)
**10. Management of Catastrophic bleeding in palliative care includes**	
**g) I don't Know**	
**h) Midazolam**	
**i) Vitamin K**	
**j) Dark towel**	
**k) Tranexamic injection in large doses.**	
**l) b & d**	
**False**	323 (82.2 %)
**True**	72 (17.8 %)
**11“Golden standard” treatment of metastatic spinal cord compression includes the following**:	
**g) I don't Know**	
**h) High dose dexamethasone**	
**i) Neurosurgical intervention**	
**j) Chemotherapy**	
**k) Radiotherapy**	
**l) All of the above**	
**False**	360 (89.1 %)
**True**	44 (10.9 %)
**12. All of the following are characteristics of oral opioid analgesics except:**	
**g) I don't Know**	
**h) Effective for localized and generalized pain**	
**i) Easily administered**	
**j) Stigma and fears associated with use**	
**k) Ceiling effect to analgesia**	
**l) None of the above**	
**False**	239 (59.2 %)
**True**	165 (40.8 %)

### Attitude of the medical students regards palliative care

Concerning the attitude of the medical students about palliative care, the results showed that the total attitude scale score for the medical students ranged from 93.0 to 121.0 with the mean score of 108.8 (SD = 8.1). The results indicate that medical students have positive attitude toward palliative care. The analysis of the subscales revealed that the highest ranked attitude subscales was Fairs/Malaise Subscale (*M =* 29.0, SD = 4.3) followed by Communication Subscale (*M = 21.4*, SD = 2.5) and Relationship Subscale (*M =* 18.3, SD = 1.6). On the other hand, the lowest reported subscale from medical students were Family as Caring Subscale (*M =* 12.1, SD = 1.2), the Care of the Family Subscale (*M =* 12.9, SD = 1.3), and Active Care Subscale (*M =* 15.1, SD = 1.71).

From a different perspective through individual item analysis (see Table 4), highest reported items were item # FC 18 “Families should be concerned about helping their dying member make the best of his/her remaining life” (*M = 4*. 6, SD = 0. 6), item # RS 21 “It is beneficial for the dying person to verbalize his/her feelings” (*M = 4.4*, SD = 0.6), and item # CTF 4 “Caring for the patient’s family should continue throughout the period of grief and bereavement” (*M = 4.4*, SD = 0.6). Conversely, the lowest reported items were item # FM 8 “I would be upset when the dying person I was caring for gave up hope of getting better” (*M =* 2.4, SD = 0.6), item # FM26 “I would be uncomfortable if I entered the room of a terminally ill person and found him/her crying” (*M =* 2.9, SD = 0.9), and item # FC 26 “I would be uncomfortable if I entered the room of a terminally ill person and found him/her crying” (*M =* 2.9, SD = 0.9) .

**Table 4:** Attitude of the medical students regards palliative care (*n*=404)

**Item number**	**Subscale/Item**	**Subscale ranking**	**Item ranking**	**Mean**	**SD**
	**The total Attitude Scale Scores**			**102.65**	**11.24**
	**Fairs/Malaise Subscale**	**1**		**26.59**	**4.28**
FM 1	Giving care to the dying person is a worthwhile experience.		5	4.17	0.75
FM 5	I would not want to care for a dying person.		11	3.87	0.95
FM 7	The length of time required giving care to a dying person would frustrate me.		16	3.57	0.58
FM 13	I would hope the person I’m caring for dies when I am not present		22	3.08	0.74
FM15	I would feel like running away when the person actually died		23	3.07	0.99
FM 14	I am afraid to become friend with a dying person.		24	3.00	0.87
FM 3	I would be uncomfortable talking about impending death with the dying person		25	2.95	0.79
FM26	I would be uncomfortable if I entered the room of a terminally ill person and found him/her crying.		28	2.88	0.93
FM 8	I would be upset when the dying person I was caring for gave up hope of getting better.		29	2.44	0.62
	**Communication Subscale**	**2**		**20.69**	**3.67**
CS 27	Dying persons should be given honest answers about their condition.		7	4.07	0.77
CS 30	It is possible for nonfamily caregivers to help patients prepare for death.		12	3.79	0.61
CS 2	Death is not the worst thing that can happen to a person.		14	3.71	0.99
CS 28	Educating families about death and dying is not a nonfamily caregiver responsibility.		17	3.45	1.06
CS 6	The nonfamily caregivers should not be the one to talk about death with the dying person.		20	3.25	0.60
CS 11	When a patient asks, “Am I dying?” I think it is best to change the subject to something cheerful		21	3.12	0.91
	**Relationship Subscale**	**3**		**16.73**	**3.00**
RS 21	It is beneficial for the dying person to verbalize his/her feelings.		2	4.40	0.56
RS 10	There are times when the dying person welcomes death.		8	4.05	0.66
RS 17	As a patient nears death, the nonfamily caregiver should withdraw from his/her involvement with the patient.		16	3.67	0.65
RS 29	Family members who stay close to a dying person often interfere with the professional’s job with the patient.		19	3.26	1.16
RS 9	It is difficult to form a close relationship with the dying person		26	2.93	0.82
	**Active Care Subscale**	**4**		**16.23**	**2.82**
AC 23	Caregivers should permit dying persons to have flexible visiting schedules.		9	3.98	0.55
AC 24	The dying person and his/her family should be the in-charge decision-makers		10	3.90	0.55
AC 19	The dying person should not be allowed to make decisions about his/her physical care.		13	3.74	0.78
AC 25	Addiction to pain relieving medication should not be a concern when dealing with a dying person		18	3.45	0.73
	**Family as Caring Subscale**	**6**		**12.44**	**2.12**
FC 18	Families should be concerned about helping their dying member make the best of his/her remaining life.		1	4.56	0.56
FC 12	The family should be involved in the physical care of the dying person.		15	3,67	0.96
FC 26	I would be uncomfortable if I entered the room of a terminally ill person and found him/her crying.		27	2.88	0.94
	**The Care of The Family Subscale**	**5**		**12.41**	**2.13**
CTF 4	Caring for the patient’s family should continue throughout the period of grief and bereavement.		3	4.38	0.61
CTF 16	Families need emotional support to accept the behavior changes of the dying person.		4	4.38	0.48
CTF 22	Care should extend to the family of the dying person.		6	4.13	0.50

### Differences in attitudes toward palliative care in relation to selected demographics

An independent –sample t-test was conducted to compare the total attitude for selected demographics for the medical students, according to levene’s test equal variances assumed was used because *P* value is larger than 0.05, so the result revealed that there was significant difference in total attitude in which female mean (M=109.97) is larger than male mean [(M=105.47); *t* (402) =-5.14, *p*=0.001]. Likely, the results indicated that there was significant difference in total attitude in which exposure to Palliative Care mean (M=298) is larger than mean for those students who did not expose to Palliative Care [(M=106); *t* (402) -6.33, *p*=0.001]. On other hand, the rest of comparisons for other variables (Academic Year, Palliative Care Course as well as Education about Palliative Care) were not statistically significant. These results are displayed in Table 5.

**Table 5:** Independent t-test for comparing selected demographics in total Attitude (*n*=404)

	**Variable**	**N**	**Mean**	**SD**	**T**^c^	**df **^d^	***P* value**
Total Attitude	Gender				-5.14^a^	402	0.001^b^
	Male	109	105.47	7.97			
	Female	295	109.97	7.76			
Total Attitude	Academic Year				-1.59^a^	402	0.114
	5^th^ year	180	108.05	8.42			
	6^th^ year	224	109.33	7.73			
Total Attitude	Exposure to Palliative Care				-6.33^a^	402	0.001^b^
	No	106	104.70	0.89			
	Yes	298	110.20	0.41			
Total Attitude	Palliative Care Course				0.301^a^	402	0.76
	No	394	108.78	8.16			
	Yes	10	108.00	0.01			
Total Attitude	Education about Palliative Care				-0.636^a^	402	0.53
	No	217	108.52	7.96			
	Yes	187	109.03	8.18			

^a^Equal variance assumed

^b^Significant at α = 0.05 (2-tailed)

^c^T-test for independent groups

^d^Degrees of freedom

## Discussion

Palliative and end of life care is one essential component for quality of care for all healthcare professionals. Such components have to be integrated early in medical education and training to ensure that medical students are adequately prepared and well-equipped with essential knowledge and skills related to palliative and end of life care practices, [19–23]. This study emphasized such topic and found that although medical students have positive attitudes toward palliative and end of life care, they severely lack the appropriate knowledge and skills to manage care and needs of patients in need for palliative and end of life care. In this study, medical students are primarily in their 5^th^ and 6^th^ year indicating that they are already well-established and received clinical training in various clinical training areas and specialties. This could explain their positive attitudes as their daily clinical practice would introduced them to an integrated aspect of care that includes patients with cancer and end of life care. The results showed that students reported being aware of palliative and end of life care and did receive some education about these topics. On the other hand, not receiving formal education aspect as part of their theoretical preparation did not enable them to identify the correct information while caring for their patients. Such a paradoxical experience of medical students in this study would jeopardize the patient's life and healthcare outcome as they will rely primarily on their personal judgment and the little acquired experience during clinical training rather that referring to solid and well-established theoretical-based training on palliative and end of life care. The results of this study do support previous reports whom asserted that medical students lack the appropriate knowledge and skills of palliative and end of life care similar results in the literature [10, 15, 16, 19, 20, 24].

Medical students are trained and educated to be the future physicians and to be responsible for managing care for their patients comprehensively. Their knowledge, skills and attitudes toward all aspect of care will shape their professional character, medical decisions, and competencies that directly affects quality of care provided to their patients [19, 20, 25, 26]. Thus, their positive attitudes toward palliative and end of life care, as it is found in this study, will positively influence their willingness and level of care provided. This, eventually, support previous efforts who have reported that medical students with positive attitudes toward palliative and end of life care are more apt to provided show knowledge and skills that positively impact their level and quality of care [21–24]. What has been noticed in this study is that medical students were positive in terms of all aspect except that related to family care. This could be due to number of factors including not receiving formal education in palliative and end of life care and depending on their personal interpretations and experiences. Such situation might indicate that medical students have not been involved in palliative and end of life care and did not practice care in their actual clinical training settings. The significant role of family in providing care for their loved one especially those suffering from end of life conditions and being part of the treatment plans have been indicated by the literature [25]. Interestingly, being negative toward family care is a real unexpected notion in the Jordan culture who is part of the Arabian culture that signifies and prioritize the role of the family in caring of the sick members [22, 26].

Although medical education is considered a long period of theoretical preparation and intensive clinical training, palliative and end of life care are not integrated into medical curricula. This has been sustained by the results of this study where most of the medical students did not have any previous palliative care course nor particular education about palliative care. Such findings have been also found in other health field such as nursing where nursing students found to lack appropriate knowledge and skills related to palliative and end of life care [2, 27, 28]. These are remarkable results in the study as those students are studying in the advanced levels of their academic programs where most of them are in their 6^th^ year which is the last year. This could explain lack of knowledge and skills related to palliative and end of life care among medical student. Lack of specialized educational instructions concerning palliative and end of life care in medical education curricula might also affect students’ preference and competency to future specialization which might adversely affect quality of palliative care provided [29].

Regarding the differences related to sociodemographic, this study found that male and female medical students were different in their attitudes toward palliative care. Female students have more favorable attitudes toward palliative care than their counterparts. This could be related to passionate type of care which female are more likely and capable to practice than male medical students which also is supported by previous international reports [29]. Gender role is one significant contributing factors that have been emphasized in previous in Jordanian studies and found to influencing quality of care outcomes [28, 30, 31].

### Implications of the study

Implications of the study finding can be helpful in: education, practices, and research. *In education*, developed through utilize up to date concerning the latest knowledge of providing palliative care for those patients who suffer from life devastating health problems or dying patients. The findings of the study urge to develop scientific material to medical students introducing main concepts related to palliative and end of life care. Moreover, the medical curricula need to be updated and the palliative and end of life care need to be addressed and introduced as part of the formal theoretical education and training. *In practice*, training programs and courses in terms of continuing medical education is required especially for novice physicians whom were not exposed to palliative and end of life care. In addition, there should be specific rotation for medical students and novice physicians to palliative care aspects. *In research*, the researcher could use the results of this study to examine further areas that need to be integrated for medical education and how can we improve attitudes of medical students and novice physicians in the field of palliative care. Replication of this study using larger and more diverse sample is highly recommended. Additionally, there is need to validate tools used to measure quality of, knowledge, skills and attitudes of medical students and compare them with other health specialties such as nursing, occupational therapist. Furthermore, including samples from different countries could reflect the impact of different cultural context on the patients’ end of life care. Using qualitative approach to obtain more in-depth understanding for palliative and end of life care practices.

## Conclusion

The study revealed that medical students lack the appropriate knowledge and skills related to palliative and end of life care. However, they still adopt positive attitudes. Palliative care is not given the priority in medical education; thus, medical students are not formally trained and educated about palliative care and end of life care. The highlighted the weak knowledge that counteract their attitudes inferring that their general practices and personal information did contribute to their positive attitudes. This also indicate that medical students are almost rich ground to learn and particle palliative care in more comprehensive and standardized methods if given the opportunity. In addition, this study highlighted the great need to update and medical undergraduate education to include palliative and end of life care earlier into their education and training. Various formed are recommended to enhance the medical education with palliative care integration such as using the high fidelity simulation. Several implications have been listed based on the findings of the present study. Applying such implications that covered different medical fields (such as medical education, practice, administration, and research) could lead to several profits for the palliative health care system, the quality of the provided care, the palliative medical performance and satisfaction as well as the health status and well-being of those patients with end of life condition.

## Limitations and recommendations

The limitation of the study is related to use of a structured self – report questionnaire that could not contribute to a comprehensive understanding compared to use of a mixed quantitative and qualitative methods. Furthermore, using a longitudinal approach to examine the impact of lack of knowledge and skills related to palliative and end of life care on practices would be more informative.

## Data Availability

The authors confirm that the data supporting the findings of this study are available within the article.
